# Restaurants in the Neighborhood, Eating Away from Home and BMI in China

**DOI:** 10.1371/journal.pone.0167721

**Published:** 2016-12-13

**Authors:** Xu Tian, Li Zhong, Stephan von Cramon-Taubadel, Huakang Tu, Hui Wang

**Affiliations:** 1 College of Economics and Management, China Center for Food Security Studies, Nanjing Agricultural University, Nanjing, China; 2 Department of Agricultural Economics and Rural Development, University of Göttingen, Göttingen, Germany; 3 Department of Epidemiology, The University of Texas MD Anderson Cancer Center, Houston, Texas, United States of America; 4 Department of Epidemiology and Biostatistics, School of Public Health, Nanjing Medical University, Nanjing, China; Shanghai Diabetes Institute, CHINA

## Abstract

**Background:**

To investigate the association between environmental risk factors, eating away from home, and increasing BMI of Chinese adults.

**Methods:**

Participants were selected from the recent four waves (2004, 2006, 2009, and 2011) of the China Health and Nutrition Survey (CHNS). 10633 participants, including 5084 men and 5549 women, were used in the analysis. 24-h dietary recall data for three consecutive days with information on the time and place of consumption were collected. Nearby restaurants were measured by the number of fast food outlets, indoor restaurants, and food stands in the neighborhood. Random effects multivariable regression was used to assess associations between these variables.

**Results:**

People living in neighborhoods with large numbers of indoor restaurants are more likely to eat away from home (p<0.05). Higher frequency of eating away from home is positively associated with BMI, but this effect is only significant for men (p<0.05). Moreover, while eating dinner or breakfast away from home contributes to BMI increase for men (p<0.05), no such association is found for lunch.

**Conclusion:**

Eating dinner and breakfast away from home is positively associated with BMI for Chinese men. Labeling energy and portion size for the dishes served in indoor restaurants is recommended in China.

## Introduction

Along with China’s rapid income growth and urbanization in the past four decades, Chinese consumers have begun eating more meals in restaurants, cafeterias, and dining halls[1–5]. The share of food eaten away from home (EAFH) in total food expenditures increased steadily from 5.0% in 1992 to 14.7% in 2000, and further to 30.0% in 2008[1, 3]. Meanwhile, China also experienced a rapid increase in the prevalence of overweight and obesity[6–8], which almost doubled from approximately 24.7% in 1991 to 44.0% in 2011[9–15]. Overweight and obesity are associated with increased risk of health problems such as diabetes, cardiovascular disease, osteoarthritis, gall bladder disease, and some cancers, some of which have become major causes of morbidity, disability and mortality in China[12–14].

In the current literature EAFH is suspected to be a cause of the rising prevalence of obesity [2, 15–26]. However, the relationship between environmental risk (number of nearby restaurants), EAFH, and health outcomes (such as increasing BMI) remains unclear. Most studies look at the association between the number of nearby restaurants and obesity [2, 15–17, 22–26]. This ignores the intermediate role of EAFH, since increasing the number of restaurants will not necessarily lead to a higher frequency of EAFH. Some studies compare the nutrition of food from different sources[19, 20, 27, 28] and conclude that EAFH foods contain higher fat and energy but lower fiber and micronutrients. However, this does not take the environmental factor into account. Others investigate the determinants of eating out in China[1, 3–5, 29] and highlight the dominant role of income growth[3, 29], rapid urbanization[30], family and individual characteristics such as the wife’s employment status[3, 31], as well as increasing accessibility to food facilities. However, these studies do not relate EAFH to health outcomes.

To fill the aforementioned gap in the current literature, we first investigate the relationship between the number of nearby restaurants and EAFH, and second estimate the contribution of EAFH to BMI. Moreover, we separate restaurants into three types: fast food restaurants, indoor restaurants, and outdoor food stands, to assess whether different types of restaurant have different effects on residents’ eating behavior. In addition, we assess the effects of different meals (breakfast, lunch, and dinner) on BMI, since individuals eat away from home differently and at different times.

## Methods

### Subjects

Participants in our study were selected from the recent four waves (2004, 2006, 2009, and 2011) of the China Health and Nutrition Survey (CHNS). The sample consists of data obtained from around 4400 households utilizing a multi-stage, random cluster strategy for 9 provinces (Liaoning, Heilongjiang, Jiangsu, Shandong, Henan, Hubei, Hunan, Guangxi, and Guizhou). Three mega cities, Beijing, Chongqing, and Shanghai, joined the survey in 2011. The survey design and methods are described in detail elsewhere[32].

**Fig 1:**
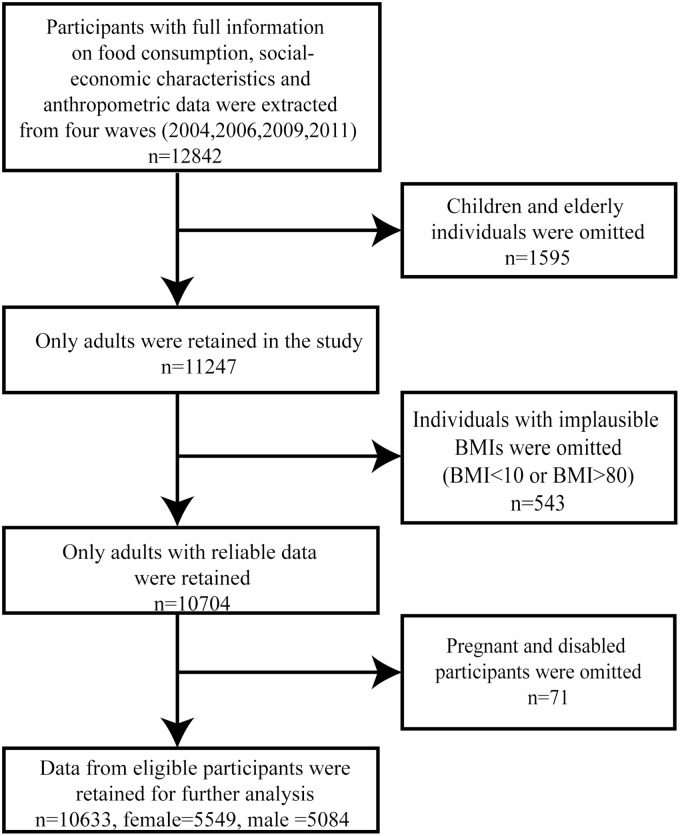
Inclusion and exclusion criteria of participants. (A) All participants are drawn from four waves of China Health and Nutrition Survey (2004, 2006, 2009, and 2011). (B) BMI refers to body mass index.

For our analysis we retained only participants with full information on food consumption (quantity, place and time), social-economics characteristics and anthropometric data (n = 12842). Pregnant and disabled participants were not considered since their body weight could not be precisely assessed (n = 71)[16]. In addition, since children and the elderly cannot be compared with adults directly, we excluded individuals below 18 and over 65 years of age (n = 1595). We also excluded individuals with implausible BMI values (BMI<10 or BMI>80) to avoid measurement error (n = 543). The remaining data from 10633 participants, 5084 men and 5549 women, were used in our analysis. These were drawn from 287 villages/communities (we have data from 188,199, 201, and 263 villages/communities in 2004, 2006, 2009, and 2011 respectively). The flow chart in Fig 1 provides information on the numbers of participants included according to the criteria outlined above.

### Data collection

#### Assessment of nearby restaurant

Numbers of restaurants are recorded in the CHNS community surveys. The neighborhood is defined as the community in urban areas and the village in rural areas. The CHNS collects data on all types of restaurant (fast food restaurants, indoor restaurants, outdoor fixed food stalls, mobile food carts, bakeries and vendors that sell breakfast) by asking community leaders how many restaurants of each type are currently operating in their communities. We classified these restaurants into three types: fast food restaurants, indoor restaurants, and outdoor food stands (including outdoor fixed food stalls, mobile food carts, bakeries and vendors that sell breakfast). Fast food restaurants are defined as restaurant chains that sell western-style food products, such as McDonald’s, Kentucky Fried Chicken and Pizza Hut. Indoor restaurants are defined as those operated inside an enclosed building with a roof and well-covered walls. Outdoor food stands include all small food stalls that are operated outdoors, such as outdoor fixed food stalls, mobile food carts that sell cooked food (e.g., dumplings, steamed bread, pancakes), bakeries and vendors that sell fried, twisted bread or other breakfast foods[2, 16]. Each type of restaurant sells characteristic kinds of food, and also reflects different availability of food due to its typical size[16]. We thus use three variables (numbers of each type of restaurant in the neighborhood) to capture the accessibility of restaurants.

#### Dietary intake

The CHNS collected details on individual food consumption (24-h recall) for three consecutive days from all participants. Data were recorded by trained interviewers through face-to-face interviews, and include the types, amounts, types of meals (breakfast, lunch, dinner, snack) and locations (home, school or the workplace, restaurant or food stand, relative’s or friend’s house, nursery school, festival or celebration) of consumption of all food items consumed[33].

#### Assessment of EAFH

For each meal (breakfast, lunch, dinner and snack) in each day, the place of consumption was reported. We define EAFH as all meals that were not consumed at home during the three survey days, including meals purchased at restaurants, fast food outlets, cafeterias and other venues such as food stands. It also includes meals that are free, hosted by friends or relatives, or are provided at the workplace.

#### Assessment of BMI

The CHNS includes anthropometric data in the physical measurement survey for all adults. The weight and height of each individual were measured by trained health workers using regularly calibrated equipment and according to the manufacturer's instructions (SECA880 scales and SECA 206 wall-mounted metal tapes). BMI was calculated as the ratio of weight (kg) divided by the square of height (m^2^). Participants were classified into four groups according to standards developed by the Chinese Working Group on Obesity in the International Life Science Association (WGOC)[34]: underweight (BMI<18.5), normal (18.5≤BMI<24), overweight (24≤BMI<28), and obese(BMI≥28). We follow the WGOC standard because Chinese people are at higher risk of obesity-related diseases or conditions at lower BMIs than Western people[35].

#### Co-variables

The CHNS also provides demographic, socioeconomic, and life style factors, including sex, age, marital status, education, employment status of household wife, per capita household income, physical activity, smoking status, and drinking status. Age is measured in years. Education is in years of formal attained education classified into three categories: primary education (years≤6), secondary education (6<years≤12), and tertiary education (years>12). The employment status of the household wife is indicated by a dichotomous variable, where 1 indicates that the wife is employed (if a woman lives in a household without men, 1 indicates that she has a job). Per capita household income was deflated to 2004 using the consumer price index (CPI). Physical activity is classified into three categories: light activity (very light and light physical activity, working in a sitting or standing position, such as office workers, sales persons, teachers), middle activity (moderate physical activity such as students, drivers, electricians, metal workers), and heavy activity (heavy and very heavy physical activity, such as farmers, dancers, miners, stonecutters). Smoking and drinking status are coded using dichotomous variables, with 1 indicating that an individual smokes or drank alcohol in the last year, respectively.

### Statistical analyses

We first studied the distribution of participants’ socioeconomic characteristics (sex, age, education, smoking and drinking status, employment and marital status, regional and yearly distribution, and physical activity) across different BMI groups for purposes of comparison.

We then measured the association between frequency of EAFH and number of nearby restaurants using multivariable linear regression. We included the employment status of the wife, marital status, sex, age, education, and per capita income in the regression model to control for the effects of these confounders. In order to control for unobserved regional variation and time-variant factors, regional dummy variables and a time trend were also included in the multivariable regression. In the next step, we investigated the association between BMI and EAFH using multivariable linear regression. Since life style factors such as physical activity, smoking and drinking status affect BMI significantly [2, 15, 16], we added these three variables to the regression model for BMI. In addition, since different types of meal (breakfast, lunch or dinner) might have different effects on BMI, we divided EAFH into three categories: breakfast, lunch, and dinner eaten away from home, respectively. Moreover, some observations were drawn from the same individual in different years, and other observations were drawn from different individuals from the same family. Such observations might be correlated with each other due to cluster effects. In order to remove this intra-group correlation, we controlled for cluster effects at household level by modifying the standard errors and the variance-covariance matrix of the estimators. Furthermore, the panel used in this study is strongly unbalanced since only a few individuals were surveyed more than once during the four waves (7842 individuals account for10633 observations). Hence, fixed effects are not appropriate because the within group variation is negligible compared with the between group variation. Random effects generalized least squares (RE) should be adopted if unobserved time-invariant individual effects exist[36]. Since the Breusch and Pagan Lagrange-multiplier (LM) test found evidence of individual effects in all models, we present only the results of the preferred RE model in the following.

All statistical analyses were performed using Stata/SE 14 (Stata Corporation, College Station, TX, USA). Regressions were run first for the total sample and then separately for men and women. The statistical significance level was set at p<0.05 (two-sided).

### Ethics

The CHNS data adopted in our research are public data from an observational study that is not a clinical trial study. The survey was approved by the review board of University of North Carolina at Chapel Hill (UNC-CH) and the Chinese Institute of Nutrition and Food Safety (INFS) at the China Center for Disease Control and Prevention (CCDC). All participants provided written informed consent and all methods were performed in accordance with the relevant guidelines and regulations. The ethics committees of the Medical Faculty of the University of Göttingen and the University of North Carolina at Chapel Hill approved our use of this data in 2013.

## Results

As described above, we extracted 12842 participants with full information from four waves of the CHNS. Children and elderly individuals (n = 1595), individuals with implausible BMI (n = 543), as well as pregnant and disabled individuals (n = 71) were omitted. We used the remaining 10633 participants in our empirical analysis.

Table 1 presents the average frequencies of EAFH during the three survey days, by BMI category. Overall, obese participants tend to eat more in restaurants or at the workplace than underweight participants. Men and younger individuals eat away from home more frequently than women and older people. Well educated participants are also more likely to eat away from home. On average, smokers and drinkers eat in restaurants or at the workplace more frequently. The data also show that employed and unmarried participants are more likely to eat away from home. Moreover, Table 1 provides evidence that EAFH might vary across regions and over years. In particular, urban citizens and participants from southern regions of China eat away from home more frequently than their rural and northern counterparts. EAFH has increased steadily over time. The last six rows of Table 1 do not reveal any clear association between EAFH and physical activity level.

**Table 1:** Frequencies of eating-away-from-home in the three-day recall period (EAFH)^a^ across personal and lifestyle characteristics as well as BMI (kg/m^2^) categories (standard errors in brackets).

BMI	underweight (<18.5)		normal (18.5~24)		overweight (24~28)		obesity (>28)		Total	
	obs.	EAFH	obs.	EAFH	obs.	EAFH	obs.	EAFH	obs.	EAFH
Total	495	1.11 (1.94)	5697	1.14 (1.99)	3378	1.14 (1.96)	1063	1.17 (2.05)	10633	1.14 (1.98)
Sex										
Men	211	0.91 (1.74)	2640	1.19 (2.01)	1720	1.41 (2.16)	513	1.46 (2.28)	5084	1.28 (2.08)
Women	284	1.26 (2.06)	3057	1.08 (1.97)	1658	0.86 (1.70)	550	0.90 (1.78)	5549	1.01 (1.88)
Age										
≤45	307	1.50 (2.11)	3024	1.38 (2.20)	1362	1.43 (2.16)	466	1.40 (2.25)	5159	1.40 (2.19)
>45	188	0.48 (1.39)	2673	0.85 (1.68)	2016	0.94 (1.79)	597	0.99 (1.87)	5474	0.89 (1.74)
Education (years)										
primary (≤6)	164	0.48 (1.39)	1720	0.62 (1.68)	986	0.71 (1.79)	303	0.71 (1.87)	3173	0.65 (1.74)
secondary (7~12)	271	1.23 (2.00)	3406	1.22 (2.04)	2088	1.22 (2.04)	667	1.27 (2.17)	6432	1.23 (2.05)
tertiary (>12)	60	2.30 (2.29)	571	2.17 (2.37)	304	1.96 (2.15)	93	1.92 (2.33)	1028	2.09 (2.30)
Currently smoking										
yes	132	0.96 (1.83)	1640	1.20 (2.01)	920	1.53 (2.32)	260	1.50 (2.42)	2952	1.32 (2.15)
no	363	1.17 (1.97)	4057	1.11 (1.98)	2458	0.99 (1.79)	803	1.06 (1.91)	7681	1.07 (1.91)
Drink last year										
yes	153	1.15 (1.95)	2000	1.29 (2.03)	1342	1.43 (2.14)	414	1.52 (2.29)	3909	1.36 (2.10)
no	342	1.10 (1.93)	3697	1.05 (1.96)	2036	0.95 (1.81)	649	0.94 (1.86)	6724	1.01 (1.91)
Employed										
yes	469	1.15 (1.97)	5102	1.17 (2.03)	2787	1.22 (2.05)	885	1.24 (2.15)	9243	1.19 (2.04)
no	26	0.46 (1.10)	595	0.84 (1.60)	591	0.75 (1.45)	178	0.80 (1.40)	1390	0.79 (1.50)
Married										
yes	386	0.92 (1.73)	5070	1.08 (1.92)	3126	1.13 (1.95)	983	1.18 (2.06)	9565	1.10 (1.94)
no	109	1.81 (2.42)	627	1.60 (2.44)	252	1.25 (2.07)	80	1.05 (2.03)	1068	1.50 (2.33)
Region										
rural	326	0.71 (1.66)	3803	0.81 (1.71)	2178	0.87 (1.78)	708	0.91 (1.83)	7015	0.84 (1.74)
urban	169	1.89 (2.18)	1894	1.78 (2.32)	1200	1.63 (2.18)	355	1.70 (2.36)	3618	1.73 (2.27)
south	355	1.20 (2.03)	3574	1.32 (2.10)	1713	1.44 (2.11)	413	1.65 (2.35)	6055	1.37 (2.12)
north	140	0.89 (1.65)	2123	0.82 (1.74)	1665	0.83 (1.74)	650	0.87 (1.78)	4578	0.83 (1.74)
Year										
2004	99	0.66 (1.59)	1074	0.88 (1.73)	547	0.95 (1.88)	148	0.80 (1.65)	1868	0.88 (1.76)
2006	87	0.90 (1.64)	1117	1.00 (1.91)	582	1.08 (1.98)	160	1.15 (2.03)	1946	1.03 (1.93)
2009	150	1.27 (2.12)	1555	1.10 (2.12)	876	0.99 (1.94)	279	1.01 (2.17)	2860	1.07 (2.07)
2011	159	1.36 (2.05)	1951	1.39 (2.03)	1373	1.34 (1.99)	476	1.38 (2.09)	3959	1.37 (2.02)
Physical activity^b^										
light	84	1.51 (1.89)	1234	1.54 (2.12)	925	1.42 (2.09)	279	1.47 (2.20)	2522	1.49 (2.11)
middle	113	1.93 (2.44)	1192	1.61 (2.29)	759	1.36 (2.15)	250	1.42 (2.19)	2,314	1.52 (2.25)
heavy	298	0.69 (1.58)	3271	0.81 (1.74)	1694	0.88 (1.76)	534	0.90 (1.87)	5797	0.83 (1.75)

Note:

^a^EAFH is defined as foods that were not consumed at home during the three survey days, including meals purchased at restaurants, fast food outlets, cafeterias and other venues such as food stands. It also includes meals that are free, hosted by friends or relatives, or are provided at the workplace.

^b^light activity (very light and light physical activity, such as office worker, sales person, teacher), middle activity (moderate physical activity such as student or driver), heavy activity (heavy and very heavy physical activity, such as farmer, dancer, loader, miner).

S1 Fig displays the frequency of EAFH by meal. The first chart shows that over 70% (72.8%) of the participants never ate away from home in 2004 during the three-day recall period of the survey. This share declined sharply to 57.0% in 2011. More specifically, the percentage of participants who had ate breakfast, lunch, and dinner away from home at least once during the three-day recall period increased, respectively, from 13.65%, 17.72% and 10.01% in 2004, to 25.46%, 28.87% and 13.21% in 2011. S1 Table provides some evidence of an increasing trend of EAFH for different types of meals. Although the average number of neighborhood restaurants declined over the survey period, the frequencies of EAFH for each meal increased steadily over time.

To study the determinants of increasing EAFH in China, we estimated multivariable regressions that explain an individual’s frequency of EAFH as a function of the number of restaurants in his/her neighborhood as well as a set of individual socio-economic and lifestyle variables. We considered three different types of restaurants, namely fast food restaurants, indoor restaurants, and outdoor food stands, as explanatory variables. Results are presented in Table 2. We find that only the number of indoor restaurants in the neighborhood is positively associated with the frequency of EAFH (p<0.05). This effect is rather small (all other things being equal, an individual who lives in a village or community with 10 more indoor restaurants will eat away from home 0.03 times more often). Fast food restaurant and outdoor food stands have no significant associations with EAFH. In addition, the wife’s employment is found to be positively associated with EAFH both for women (p<0.01) and men (p = 0.06). Moreover, results also show that women eat less in restaurants and at the workplace after marriage (p<0.01), and men eat more frequently away from home than women (p<0.01). Education and income are found to be positively correlated with the frequency of EAFH (all p<0.01). Significant regional difference are found, with urban citizens and southerners displaying significantly higher frequencies of EAFH (all p<0.01). A significantly increasing trend of EAFH is also detected (all p<0.05).

**Table 2:** Association between number of nearby restaurants and eating away from home (EAFH).

Dependent variable: EAFH	Total		Men		Women	
	Coefficient	S.E.	Coefficient	S.E.	Coefficient	S.E.
Fast food	-0.004	(0.005)	-0.003	(0.007)	-0.005	(0.005)
Indoor restaurant	0.003	(0.001)*	0.005	(0.002)*	0.002	(0.001)
Outdoor food stand	-0.001	(0.001)	-0.002	(0.001)	-0.001	(0.001)
Wife employed	0.200	(0.050)**	0.130	(0.065)*	0.256	(0.061)**
Married	-0.211	(0.074)**	-0.096	(0.108)	-0.352	(0.099)**
Male	0.290	(0.037)**				
Old adult	-0.445	(0.042)**	-0.399	(0.059)**	-0.496	(0.050)**
Secondary education	0.294	(0.041)**	0.300	(0.063)**	0.300	(0.053)**
Tertiary education	0.750	(0.091)**	0.754	(0.119)**	0.732	(0.119)**
ln(Income)	0.173	(0.026)**	0.240	(0.035)**	0.113	(0.031)**
Rural	-0.712	(0.056)**	-0.737	(0.072)**	-0.691	(0.069)**
South	0.564	(0.046)**	0.569	(0.061)**	0.567	(0.052)**
Time trend	0.084	(0.018)**	0.085	(0.026)**	0.085	(0.022)**
Constant	-0.778	(0.264)**	-1.278	(0.347)**	-0.069	(0.313)
Observations	10633		5084		5549	
BP-LM test	122.78**		46.01**		81.25**	

Notes: EAFH is defined as the frequency of EAFH during the survey recall period. S.E. refers to the standard error, ** p<0.01; * p<0.05. Independent variables included: three types of restaurants (number of these restaurants in the neighborhood); secondary education (6~12 years), tertiary education (>12 years), ln(Income) (logarithm of income deflated to 2004), time trend (1 = 2004, 2 = 2006, 3 = 2009, 4 = 2011), old adult (age>45), wife employed, married, male, rural, and south are dichotomous variables where 1 refers to the control group and 0 to the reference group. Rejection of BP-LM test indicates that random effect multivariable regression model is preferred.

S2 Fig presents the changing BMI distribution over years by sex. Almost half (49.65%) of the male participants in 2011 were considered overweight or obese according to WGOC standards, compared with 36.47% in 2004. The share of overweight and obese women also increased sharply, from 37.70% in 2004 to 43.55% in 2011.

The increasing prevalence of overweight and obesity is a result of various factors. Table 3 presents corresponding multivariable regression results. EAFH is significantly associated with BMI increase for men (p<0.01), but not for women (p = 0.88).

**Table 3:** Association between eating away from home (EAFH) and BMI (I).

Dependent variable: BMI	Total		Men		Women	
	Coefficient	S.E.	Coefficient	S.E.	Coefficient	S.E.
EAFH	0.040	(0.015)**	0.070	(0.021)**	0.003	(0.020)
Heavy activity	-0.243	(0.093)**	-0.556	(0.138)**	0.021	(0.127)
Middle activity	-0.021	(0.095)	-0.191	(0.137)	0.152	(0.126)
Married	0.794	(0.139)**	0.896	(0.153)**	0.672	(0.227)**
Male	0.402	(0.104)**				
Old adult	0.696	(0.072)**	0.255	(0.110)*	1.052	(0.098)**
Secondary education	0.069	(0.082)	0.616	(0.121)**	-0.278	(0.117)*
Tertiary education	-0.466	(0.153)**	0.385	(0.219)	-1.239	(0.209)**
ln(Income)	0.084	(0.039)*	0.141	(0.056)*	0.024	(0.052)
Smoking	-0.445	(0.108)**	-0.395	(0.115)**	-0.356	(0.342)
Drinking	0.161	(0.080)*	0.197	(0.095)*	0.115	(0.148)
Rural	-0.059	(0.093)	0.030	(0.127)	-0.221	(0.129)
South	-1.325	(0.085)**	-1.328	(0.118)**	-1.334	(0.113)**
Time trend	0.238	(0.030)**	0.243	(0.036)**	0.239	(0.042)**
Constant	21.730	(0.417)**	21.274	(0.622)**	22.518	(0.554)**
Observations	10633		5084		5549	
BP-LM test	1827.62**		620.33**		1154.30**	

Notes: BMI is the ratio of weight divided by square of height (kg/m^2^). S.E. refers to the standard error, ** p<0.01; * p<0.05. Independent variables included: EAFH (frequency of EAFH during the survey recall period), light activity (very light and light physical activity, such as office worker, sales person, teacher), middle activity (moderate physical activity such as student or driver), heavy activity (heavy and very heavy physical activity, such as farmer, dancer, loader, miner).educational level (primary education-reference group, secondary education or tertiary education), ln(income) (logarithm of income deflated to 2004), time trend (1 = 2004, 2 = 2006, 3 = 2009, 4 = 2011), smoking (1 = currently smoking), drinking (1 = drank last year), old adult (age>45), wife employed, married, male, rural, and south are dichotomous variables where 1 refers to the control group and 0 to the reference group. Rejection of BP-LM test indicates that random effect multivariable regression model is preferred.

Table 4 reports the results of a model in which we include the numbers of breakfasts, lunches and dinners eaten away from home separately, rather than the total number of EAFH. We find that only dinner eaten away from home is significantly correlated with BMI (p<0.01), and that the impact is modest (eating one more dinner away from home in the three-day recall period is associated with a 0.17 increase in BMI). However, we find divergent results for women and men. Particularly, eating breakfast and dinner away from home both have significant positive effects on men’s BMI (p = 0.03 and p = 0.04 respectively), while eating lunch away from home is not associated with increasing BMI (p = 0.60). Eating away from home is not associated with BMI increase for women, and we even find some evidence that women who eat lunch away from home more often have lower BMI (p = 0.40). Table 4 also shows that marital status, age, and region affect BMI for both men and women. In particular, married adults, older individuals, and those who live in northern regions have significantly higher BMI than their counterparts (all p<0.05). Interestingly, education, income, and lifestyle factors such as smoking and drinking have different effects on BMI for men and women. For instance, well-educated and high-income men have higher BMIs than men with lower education and income (all p<0.05), but women with higher education level are found to have lower BMI (all p<0.05). Moreover, smoking is negatively associated with the BMI of men (p<0.01) but not of women (p = 0.28), while drinking is associated with higher BMI for men (p = 0.04) but has no significant effect for women (p = 0.44). Finally, no significant disparity is found between rural and urban respondents in terms of BMI (all p>0.05), but BMI has increased significantly over time (p<0.01).

**Table 4:** Association between eating away from home (EAFH) and BMI (II).

Dependent variable: BMI	Total		Men		Women	
	Coefficient	S.E.	Coefficient	S.E.	Coefficient	S.E.
Breakfast	0.06	(0.032)	0.091	(0.042)*	0.007	(0.049)
Lunch	-0.039	(0.035)	0.026	(0.049)	-0.109	(0.052)*
Dinner	0.166	(0.060)**	0.154	(0.075)*	0.176	(0.094)
Heavy activity	-0.231	(0.093)*	-0.547	(0.137)**	0.033	(0.127)
Middle activity	-0.015	(0.095)	-0.184	(0.137)	0.156	(0.127)
Married	0.799	(0.139)**	0.904	(0.153)**	0.669	(0.227)**
Male	0.405	(0.104)**				
Old adult	0.692	(0.072)**	0.254	(0.110)*	1.044	(0.099)**
Secondary education	0.067	(0.082)	0.609	(0.122)**	-0.274	(0.117)*
Tertiary education	-0.445	(0.152)**	0.388	(0.219)	-1.19	(0.211)**
ln(income)	0.088	(0.039)*	0.142	(0.056)*	0.03	(0.052)
Smoking	-0.448	(0.108)**	-0.397	(0.115)**	-0.364	(0.340)
Drinking	0.16	(0.080)*	0.195	(0.095)*	0.114	(0.147)
Rural	-0.072	(0.093)	0.021	(0.127)	-0.237	(0.130)
South	-1.335	(0.085)**	-1.34	(0.119)**	-1.338	(0.114)**
Time trend	0.238	(0.030)**	0.242	(0.037)**	0.241	(0.042)**
Constant	21.697	(0.416)**	21.276	(0.622)**	22.467	(0.552)**
Observations	10633		5084		5549	
BP-LM test	1834.98**		622.42**		1159.94**	

Notes: BMI is the ratio of weight divided by square of height (kg/m^2^). S.E. refers to the standard error, ** p<0.01; * p<0.05. Independent variables included: breakfast/lunch/dinner (number of times breakfast/lunch/dinner were eaten away from home during the survey recall period), light activity (very light and light physical activity, such as office worker, salesperson, teacher), middle activity (moderate physical activity such as student or driver), heavy activity (heavy and very heavy physical activity, such as farmer, dancer, loader, miner).educational level (primary education-reference group, secondary education or tertiary education), ln(income) (logarithm of income deflated to 2004), time trend (1 = 2004, 2 = 2006, 3 = 2009, 4 = 2011), smoking (1 = currently smoking), drinking (1 = drank last year), old adult (age>45), wife employed, married, male, rural, and south are dichotomous variables where 1 refers to the control group and 0 to the reference group. Rejection of BP-LM test indicates that random effect multivariable regression model is preferred.

To test the robustness of our findings, we conducted the two types of sensitivity analysis: first, we estimated our model separately for rural and urban regions; second, we included total calorie intake as an additional explanatory variable in the model that investigates the association between EAFH and BMI. Results are presented in S2 and S3 Tables. We find that results differ between rural and urban areas, but the key finding, that men are more likely to be influenced by EAFH, is not affected. In particular, eating dinner or breakfast away from home is associated with increased BMI for males. Moreover, only indoor restaurants in the neighborhood are positively correlated with EAFH, but this effect is only statistically significant in urban areas.

## Discussion

Using a large sample of 10633 men and women aged 18–65 years from 4 waves CHNS (2004, 2006, 2009, 2011) data, we investigate the relationship between the number of nearby restaurants, EAFH, and BMI for Chinese people. Three important findings are detected. First, results indicate that the frequency of EAFH is positively associated with the number of indoor restaurants, but not significantly associated with the numbers of outdoor food stands and fast food restaurants. Second, EAFH is positively associated with BMI, but only for men. Third, only eating dinner or breakfast away from home is significantly correlated with increased BMI for men, whereas eating lunch away from home has no significant effect.

Numerous studies have investigated the undergoing nutrition transition in China. This transition is characterized by a shift from traditional Chinese diets to modern western diets, and changing lifestyles such as the increasing frequency of EAFH[2, 5, 37–39]. However, increasing the number of restaurants in a community (village)will not directly increase peoples’ BMIs if they do not eat at these places. Indoor restaurants in China are typically full-service restaurants which provide diversified dishes[3]. They also play an important role in social activity[2]. Indoor restaurants near home are often used to meet friends, relax after work, and enjoy diverse dishes. Therefore, the availability of more indoor restaurants in the neighborhood might stimulate residents’ EAFH. In contrast, fast food restaurants usually offered limited, low-cost fast food, good for breakfast and lunch during working hours but not for formal meals. Hence fast food restaurants will probably increase EAFH by people who work but do not necessarily live nearby, and also be mainly located in business areas. Moreover, fast food restaurants are more popular with young people, while adults preferred to eat at indoor restaurants in China[16]. Since our study only looked at the effect of the availability of neighborhood restaurants on adults (i.e. individuals aged under18 years are not considered), our finding of no association between fast food restaurants and EAFH is not unexpected. On the other hand, outdoor food stands mainly provide simple Chinese dishes, such as porridge, steamed bread, deep-fried dough sticks, and pancakes for breakfast, or luncheonette foods such as dumplings, noodles, fried rice and rice topped with dishes. These foods are characterized by convenience, low price, limited choices and low nutritional value[2]. Therefore, outdoor food stands tend to be favored by poor rural-urban migrants. These migrants are not included in our survey data, which might explain why no significant association between the number of fast food restaurants and EAFH could be detected.

EAFH is commonly suspected to increase the risk of overweight and obesity. A systematic review indicates that the nutritional quality of foods consumed away from home is much poorer than that of foods prepared at home due to their higher fat and energy content combined with lower fiber and less micronutrients[27]. Similar conclusion can also be drawn from our data (see the S4 Table). Numerous studies have found positive associations between eating at restaurants and BMI increase. A study among 10 European countries by the European Prospective Investigation into Cancer and Nutrition (EPIC) study finds that eating at restaurants was positively associated with BMI for men (p = 0.003) but not for women, consistent with our results. However, no association is reported between BMI and eating at work for both sexes[15]. That might explain the insignificant association between eating lunch away from home and BMI found in our study, since employers in China typically offer free or low-cost lunches or food coupons to employees during working hours for use at company-owned restaurants, cafeterias, and food shops[3]. Therefore, eating lunch and dinner away from home could have quite different effects on BMI since the former usually takes place at the workplace or nearby, whereas the latter usually occurs in formal restaurants, especially indoor restaurants. One recent study on the association between restaurants and BMI in rural China using CHNS data also indicates that indoor restaurants in the neighborhood contributed to BMI increase for men, while outdoor food stalls and fast food restaurants are associated with BMI decline[2]. Another study investigates the dynamic relations between fast-food restaurants and body weight status in China using past increases in the numbers of fast food restaurants to measure changes in the local food environment. Results show that the number of western fast food restaurants is positively associated with subsequent increases in waist-to-height and waist-to-hip ratios for rural people[16]. The inconsistency between our results and theirs might be caused by different obesity measurement and methodologies used for analysis. On the other hand, deep fried foods such as fried twisted bread (You tiao), fried dumplings (Jiao zi), and fried doughnuts are very popular for breakfast in China, especially for men. These foods are very oily and not so convenient to prepare at home. This could explain the positive association between eating breakfast away from home and increasing BMI for men.

In addition, other confounders such as socioeconomic factors are also related with EAFH and BMI. For instance, the wife’s employment status, which is widely used as an indicator of the opportunity cost of time, increases the frequency of EAFH in our results, consistent with expectations and previous studies[5]. Married women eat more at home, while men’s eating habits are not affected by marital status. Men display higher frequencies of EAFH since Chinese men engage in more social activities which usually take place in restaurants[2]. Older individuals(age>45) are more likely to eat at home, while well-educated and high-income individuals tend to EAFH more frequently, consistent with findings in China and other countries[1, 3, 4, 40, 41]. Urban citizens and respondents in southern regions tend to EAFH more frequently than their rural and northern counterparts. That might be caused by differences in lifestyle. In addition, an increasing trend of EAFH is detected, consistent with the facts revealed in other studies[2, 29]. The determinants of BMI besides EAFH that we detected are also similar to previous studies. Adults with heavy physical activity have lower BMIs, but the association is only significant for men. That might be because most women work in sectors that require only light or moderate activity. Married and older adults have higher BMI values than unmarried and young people as revealed in other studies[2, 41]. Well-educated and high-income men have higher BMIs[41], while women with higher education and incomes care more about their body figure and have more knowledge of nutrition and intention to control their weights[2]. In addition, smoking is associated with lower BMI[41], while drinking increases BMI, all were only significant among men[2]. We find that respondents from southern regions have lower BMI than their northern counterparts, which is consistent with previous findings[10], but we do not find significant rural-urban differences. Finally, we also confirm that BMI has been increasing over time.

We contribute to the current literature in several respects. First, we bridge the relationship between environmental risk factors (number of restaurants in the neighborhood), eating behavior (EAFH), and health outcomes (increasing BMI). Second, we conduct our analyses separately for men and women and find different results. Third, we divide EAFH by type of meal, and results indicate that only eating dinner or breakfast away from home increases BMI.

This study has several limitations. First, information on the type of restaurant each meal was consumed in could further clarify the relationship between environmental risk factors, consumer behavior, and health outcome. However this information is not available. Future research could address this gap. Second, a consumer’s dietary decision might not only be affected by the restaurants in his or her neighborhood, but also by restaurants that are a bit farther away, such as well-known restaurants serving famous local dishes. Ignoring food consumption in restaurants outside the immediate neighborhood would lead us to underestimate the effect of restaurants on BMI. This limitation could not be solved in our study due to data availability. Third, eating in restaurants and companies’ canteens might have different effects on BMI due to the different dishes served, which however could not be distinguished in our data. Finally, our findings only refer to correlation rather than causality, since rising BMI and related dietary habits might also be creating more demand for EAFH and restaurants.

In conclusion, we find that different types of restaurants have very different effects on dietary consumption, and that only indoor restaurants in the neighborhood are positively correlated with EAFH. Moreover, the effects of eating out on BMI differ by sex: only men are found to be significantly influenced by EAFH. Furthermore, our results also show that only dinner and breakfast eaten away from home are positively associated with men’s BMI.

Our results indicate that increasing frequency of eating dinner or breakfast in indoor restaurants might be correlated with China’s raising prevalence of overweight and obesity. Labeling energy and portion size for the dishes served in indoor restaurants might help reducing over-eating and contribute to controlling body mass, particularly for men who frequently EAFH for social reasons.

## Supporting Information

S1 Fig The frequency of eating away from home by different meal. The upper left panel shows the total frequency of eating out during the three-day recall period. The upper right panel shows the eating out frequency of breakfast, and the lower left and right panels show the eating out frequency of lunch and dinner, respectively. White indicates2004, light grey 2006, dark grey 2009, and black 2011.
(DOCX)

S2 Fig The distribution of BMI among male and female respondents. The left and right panels show the BMI distributions over time for male and female respondents, respectively. White indicates 2004, light grey 2006, dark grey 2009, and black 2011.
(DOCX)

S1 Table Numbers of different types of restaurants & meals away from home over time. Note: EAFH is defined as meals that were not consumed at home during the three survey days, including meals purchased at restaurants, fast food outlets, cafeterias and other venues such as food stands. It also includes meals that are free, hosted by friends or relatives, or are provided at the workplace. Values in brackets are standard deviation. Summary: Results show that numbers of restaurants in the neighborhood was declining over time, especially small restaurants. On the other hand, numbers of meals eating away from home increased steadily over time.
(DOCX)

S2 Table Association between number of nearby restaurants and eating away from home (EAFH) for rural and urban regions. Notes: EAFH is the frequency of EAFH (It is defined as meals that were not consumed at home during the three survey days, including meals purchased at restaurants, fast food outlets, cafeterias and other venues such as food stands. It also includes meals that are free, hosted by friends or relatives, or are provided at the workplace.). BMI is the ratio of weight divided by square of height (kg/m^2^). Values in brackets are standard errors, ** p<0.01; * p<0.05. Coefficients in the first model (upper three rows) are estimated using multivariable linear regression models by adjusting employment status of household wife, marital status, sex, regional dummy (south or north), age, education, and income. Coefficients in the last two model (association between BMI and EAFH) are estimated using multivariable linear regression models by adjusting physical activity level, employment status of household wife, marital status, sex, regional dummy (south or north), age, education, smoking and drinking status and income. Summary: Results show that men are more likely to be influenced by EAFH. In particular, eating dinner away from home is a risk factor of increasing BMI. Moreover, only indoor restaurants in the neighborhood were positively correlated with people’s EAFH, but it is only statistically significant in urban area.
(DOCX)

S3 Table Association between EAFH and BMI after adjusted by total calorie intake. Notes: EAFH is the frequency of EAFH (It is defined as meals that were not consumed at home during the three survey days, including meals purchased at restaurants, fast food outlets, cafeterias and other venues such as food stands. It also includes meals that are free, hosted by friends or relatives, or are provided at the workplace.). BMI is the ratio of weight divided by square of height (kg/m2). Values in brackets are standard errors, ** p<0.01; * p<0.05. Coefficients are estimated using multivariable linear regression models by adjusting total calorie intake, physical activity level, employment status of household wife, marital status, sex, regional dummy (south or north), age, education, smoking and drinking status and income. Summary: Results show that men are more likely to be influenced by EAFH. In particular, eating dinner away from home is a risk factor of increasing BMI.
(DOCX)

S4 Table Percentage of energy derived from each food eaten at different locations. Summary: Results show that individuals eat more mushrooms, livestock, poultry and snacks, and drink more beverages and alcohol when they eat away from home. Vegetables, fruit, dairy, eggs and fast food are consumed less frequently away from home.
(DOCX)
