# Supplementary material for: Restaurants in the Neighborhood, Eating Away from Home and BMI in China
Source: PLoS One. 2016 Dec 13;11(12):e0167721. doi: 10.1371/journal.pone.0167721 (PMC5154538; doi:10.1371/journal.pone.0167721)

**Title:** Restaurants in the neighborhood, eating away from home and BMI in China

**Authors:**Xu Tian^a^, Li Zhong^a^, Stephan von Cramon-Taubadel^b^, Huakang Tu^c^, Hui Wang^d*^

**Supporting Figure 1. The frequency of eating away from home by different meal.**

The upper left panel shows the total frequency of eating out during the three-day recall period. The upper right panel shows the eating out frequency of breakfast, and the lower left and right panels show the eating out frequency of lunch and dinner, respectively. White indicates 2004, light grey 2006, dark grey 2009, and black 2011.


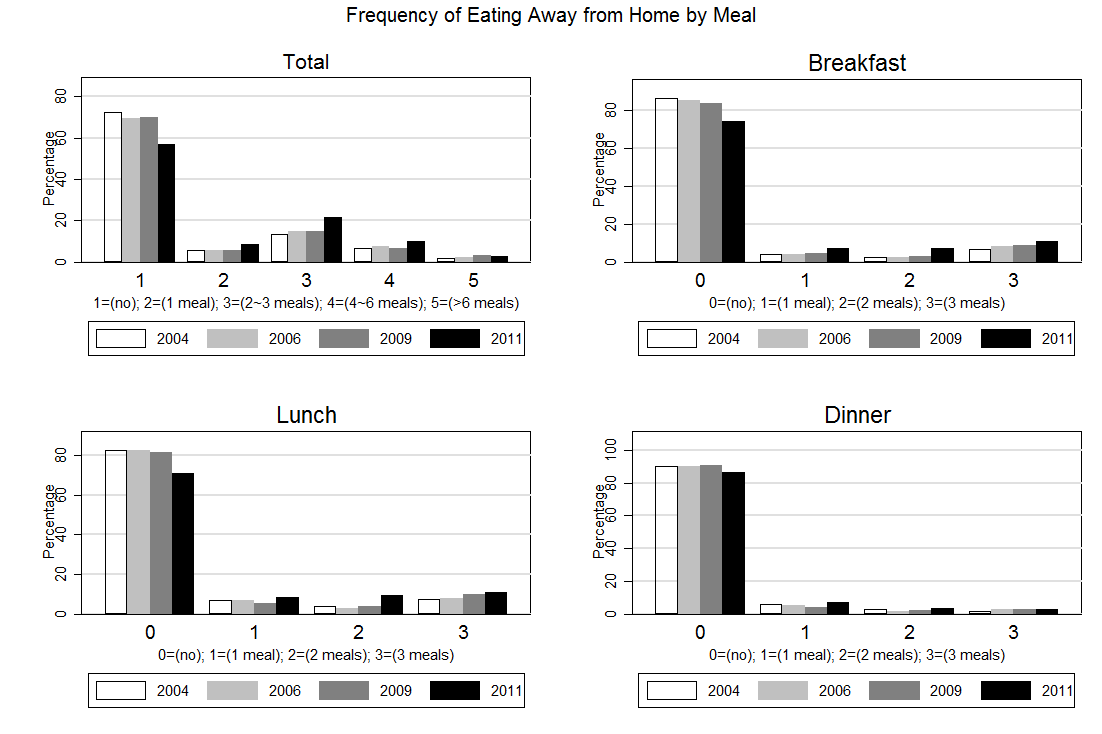

Supplement: S1 Fig — The upper left panel shows the total frequency of eating out during the three-day recall period. The upper right panel shows the eating out frequency of breakfast, and the lower left and right panels show the eating out frequency of lunch and dinner, respectively. White indicates2004, light grey 2006, dark grey 2009, and black 2011. (DOCX) [file pone.0167721.s001.docx]
