# Supplementary material for: Restaurants in the Neighborhood, Eating Away from Home and BMI in China
Source: PLoS One. 2016 Dec 13;11(12):e0167721. doi: 10.1371/journal.pone.0167721 (PMC5154538; doi:10.1371/journal.pone.0167721)

**Title:** Restaurants in the neighborhood, eating away from home and BMI in China

**Authors:**Xu Tian^a^, Li Zhong^a^, Stephan von Cramon-Taubadel^b^, Huakang Tu^c^, Hui Wang^d*^

**Supporting Figure 2. The distribution of BMI among male and female** **respondents.**

The left and right panels show the BMI distributions over time for male and female respondents, respectively. White indicates 2004, light grey 2006, dark grey 2009, and black 2011.


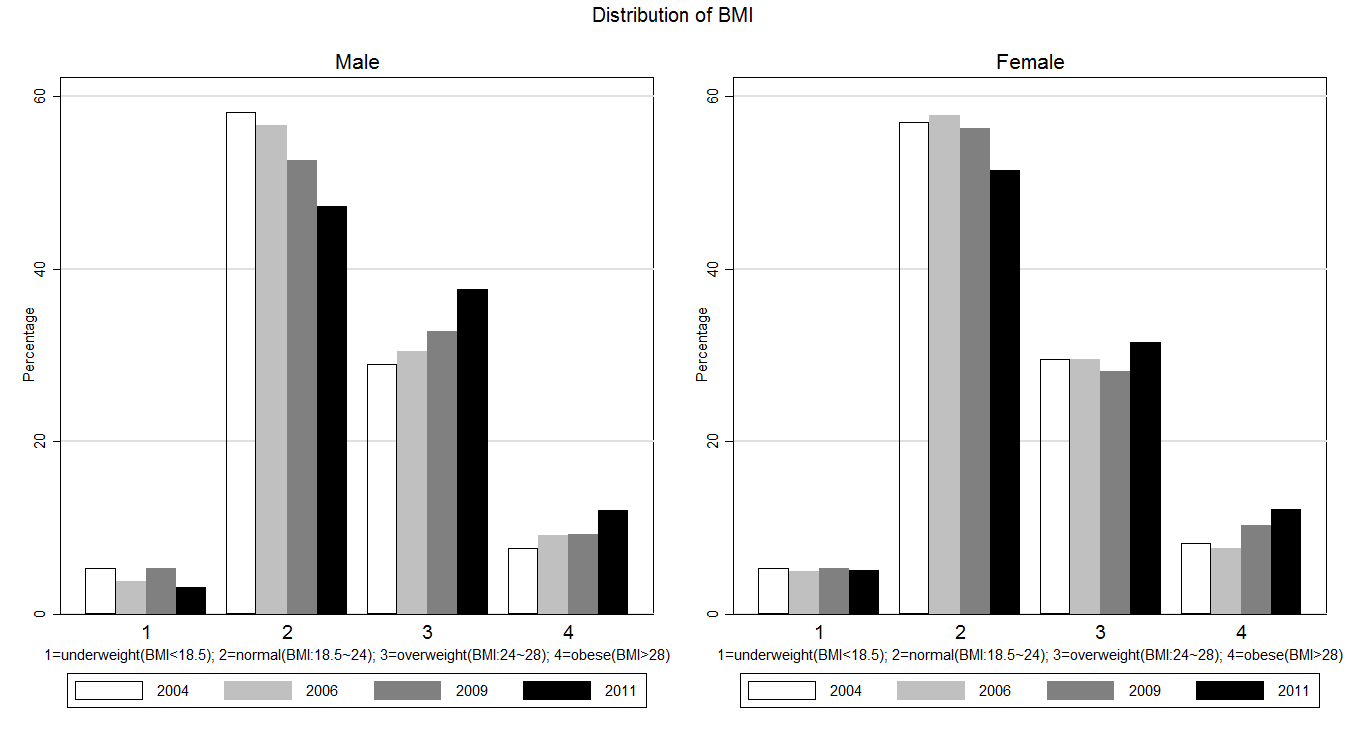

Supplement: S2 Fig — The left and right panels show the BMI distributions over time for male and female respondents, respectively. White indicates 2004, light grey 2006, dark grey 2009, and black 2011. (DOCX) [file pone.0167721.s002.docx]
