# Supplementary material for: Restaurants in the Neighborhood, Eating Away from Home and BMI in China
Source: PLoS One. 2016 Dec 13;11(12):e0167721. doi: 10.1371/journal.pone.0167721 (PMC5154538; doi:10.1371/journal.pone.0167721)
Supplement: S1 Table — Note: EAFH is defined as meals that were not consumed at home during the three survey days, including meals purchased at restaurants, fast food outlets, cafeterias and other venues such as food stands. It also includes meals that are free, hosted by friends or relatives, or are provided at the workplace. Values in brackets are standard deviation. Summary: Results show that numbers of restaurants in the neighborhood was declining over time, especially small restaurants. On the other hand, numbers of meals eating away from home increased steadily over time. (DOCX) [file pone.0167721.s003.docx]

**Title:** Restaurants in the neighborhood, eating away from home and BMI in China

**Authors:**Xu Tian^a^, Li Zhong^a^, Stephan von Cramon-Taubadel^b^, Huakang Tu^c^, Hui Wang^d*^

**Supporting Table 1. Numbers of different types of restaurants & meals away from home over time.**

| Year | 2004 |  | 2006 |  | 2009 |  | 2011 |  | Total |
| --- | --- | --- | --- | --- | --- | --- | --- | --- | --- |
|  | (n=1868) |  | (n=1946) |  | (n=2860) |  | (n=3959) |  | (n=10633) |
| Type of restaurants | 33.16 |  | 31.49 |  | 22.29 |  | 24.45 |  | 26.69 |
|  | (53.25) |  | (42.12) |  | (35.62) |  | (36.71) |  | (41.04) |
| Fast food | 0.42 |  | 0.57 |  | 0.15 |  | 0.87 |  | 0.54 |
|  | (1.90) |  | (2.90) |  | (0.58) |  | (6.00) |  | (3.97) |
| Indoor restaurants | 12.23 |  | 10.93 |  | 8.93 |  | 8.92 |  | 9.87 |
|  | (32.78) |  | (16.64) |  | (14.72) |  | (14.33) |  | (19.38) |
| Outdoor food stands | 20.50 |  | 19.99 |  | 13.21 |  | 14.66 |  | 16.27 |
|  | (30.05) |  | (28.87) |  | (24.05) |  | (25.79) |  | (26.89) |
| EAFH | 0.88 |  | 1.03 |  | 1.07 |  | 1.37 |  | 1.14 |
|  | (1.76) |  | (1.93) |  | (2.07) |  | (2.02) |  | (1.98) |
| breakfast_away | 0.30 |  | 0.33 |  | 0.37 |  | 0.54 |  | 0.42 |
|  | (0.82) |  | (0.87) |  | (0.91) |  | (1.02) |  | (0.94) |
| lunch_away | 0.36 |  | 0.36 |  | 0.42 |  | 0.61 |  | 0.47 |
|  | (0.86) |  | (0.87) |  | (0.95) |  | (1.05) |  | (0.97) |
| dinner_away | 0.16 |  | 0.17 |  | 0.17 |  | 0.22 |  | 0.19 |
|  | (0.52) |  | (0.58) |  | (0.61) |  | (0.63) |  | (0.60) |

Note: EAFH is defined as meals that were not consumed at home during the three survey days, including meals purchased at restaurants, fast food outlets, cafeterias and other venues such as food stands. It also includes meals that are free, hosted by friends or relatives, or are provided at the workplace. Values in brackets are standard deviation.

Summary: Results show that numbers of restaurants in the neighborhood was declining over time, especially small restaurants. On the other hand, numbers of meals eating away from home increased steadily over time.
