# Supplementary material for: Restaurants in the Neighborhood, Eating Away from Home and BMI in China
Source: PLoS One. 2016 Dec 13;11(12):e0167721. doi: 10.1371/journal.pone.0167721 (PMC5154538; doi:10.1371/journal.pone.0167721)
Supplement: S2 Table — Notes: EAFH is the frequency of EAFH (It is defined as meals that were not consumed at home during the three survey days, including meals purchased at restaurants, fast food outlets, cafeterias and other venues such as food stands. It also includes meals that are free, hosted by friends or relatives, or are provided at the workplace.). BMI is the ratio of weight divided by square of height (kg/m2). Values in brackets are standard errors, ** p<0.01; * p<0.05. Coefficients in the first model (upper three rows) are estimated using multivariable linear regression models by adjusting employment status of household wife, marital status, sex, regional dummy (south or north), age, education, and income. Coefficients in the last two model (association between BMI and EAFH) are estimated using multivariable linear regression models by adjusting physical activity level, employment status of household wife, marital status, sex, regional dummy (south or north), age, education, smoking and drinking status and income. Summary: Results show that men are more likely to be influenced by EAFH. In particular, eating dinner away from home is a risk factor of increasing BMI. Moreover, only indoor restaurants in the neighborhood were positively correlated with people’s EAFH, but it is only statistically significant in urban area. (DOCX) [file pone.0167721.s004.docx]

**Title:** Restaurants in the neighborhood, eating away from home and BMI in China

**Authors:**Xu Tian^a^, Li Zhong^a^, Stephan von Cramon-Taubadel^b^, Huakang Tu^c^, Hui Wang^d*^

**Supporting Table 2. Association between number of nearby restaurants and eating away from home (EAFH) for rural and urban regions.**

|  | Urban sample | | | | | |  | Rural sample | | | | | |
| --- | --- | --- | --- | --- | --- | --- | --- | --- | --- | --- | --- | --- | --- |
|  | Total |  | Male |  | Female |  |  | Total |  | Male |  | Female |  |
| Association between EAFH and restaurants | | | |  |  |  |  |  |  |  |  |  |  |
| Fast food | -0.035 | (0.017)* | -0.048 | (0.026) | -0.026 | (0.017) |  | -0.002 | (0.005) | -0.001 | (0.007) | -0.002 | (0.005) |
| Indoor restaurants | 0.008 | (0.003)* | 0.008 | (0.004)* | 0.008 | (0.004)* |  | 0.002 | (0.001) | 0.003 | (0.003) | 0.001 | (0.001) |
| Outdoor food stands | -0.001 | (0.002) | -0.001 | (0.003) | -0.001 | (0.003) |  | -0.002 | (0.001) | -0.002 | (0.001) | -0.002 | (0.001) |
| Association between BMI and EAFH (I) | | | |  |  |  |  |  |  |  |  |  |  |
| EAFH | 0.035 | (0.026) | 0.061 | (0.038) | -0.019 | (0.033) |  | 0.048 | (0.017)** | 0.068 | (0.025)** | 0.021 | (0.025) |
| Association between BMI and EAFH (II) | | | |  |  |  |  |  |  |  |  |  |  |
| Breakfast | 0.035 | (0.052) | 0.000 | (0.068) | 0.042 | (0.074) |  | 0.088 | (0.041)* | 0.134 | (0.053)* | 0.001 | (0.062) |
| Lunch | -0.024 | (0.058) | 0.056 | (0.077) | -0.137 | (0.080) |  | -0.009 | (0.044) | -0.002 | (0.062) | -0.014 | (0.057) |
| Dinner | 0.255 | (0.103)* | 0.271 | (0.124)* | 0.191 | (0.148) |  | 0.075 | (0.062) | 0.056 | (0.090) | 0.085 | (0.080) |

Notes: EAFH is the frequency of EAFH (It is defined as meals that were not consumed at home during the three survey days, including meals purchased at restaurants, fast food outlets, cafeterias and other venues such as food stands. It also includes meals that are free, hosted by friends or relatives, or are provided at the workplace.). BMI is the ratio of weight divided by square of height (kg/m^2^). Values in brackets are standard errors, ** p<0.01; * p<0.05. Coefficients in the first model (upper three rows) are estimated using multivariable linear regression models by adjusting employment status of household wife, marital status, sex, regional dummy (south or north), age, education, and income. Coefficients in the last two model (association between BMI and EAFH) are estimated using multivariable linear regression models by adjusting physical activity level, employment status of household wife, marital status, sex, regional dummy (south or north), age, education, smoking and drinking status and income.

Summary: Results show that men are more likely to be influenced by EAFH. In particular, eating dinner away from home is a risk factor of increasing BMI. Moreover, only indoor restaurants in the neighborhood were positively correlated with people’s EAFH, but it is only statistically significant in urban area.
