# Supplementary material for: Restaurants in the Neighborhood, Eating Away from Home and BMI in China
Source: PLoS One. 2016 Dec 13;11(12):e0167721. doi: 10.1371/journal.pone.0167721 (PMC5154538; doi:10.1371/journal.pone.0167721)
Supplement: S4 Table — Summary: Results show that individuals eat more mushrooms, livestock, poultry and snacks, and drink more beverages and alcohol when they eat away from home. Vegetables, fruit, dairy, eggs and fast food are consumed less frequently away from home. (DOCX) [file pone.0167721.s006.docx]

**Title:** Restaurants in the neighborhood, eating away from home and BMI in China

**Authors:**Xu Tian^a^, Li Zhong^a^, Stephan von Cramon-Taubadel^b^, Huakang Tu^c^, Hui Wang^d*^

**Supporting Table 4.** Percentage of energy derived from each food eaten at different locations

| Food | Individuals at least eat away from home once | | | |  | All individuals | | | |
| --- | --- | --- | --- | --- | --- | --- | --- | --- | --- |
|  | EAFH | Restaurant | Work | Home |  | EAFH | Restaurant | Work | Home |
| S taple | 24.17% | 14.06% | 10.11% | 75.83% |  | 8.71% | 5.07% | 3.64% | 91.29% |
| Starch | 26.77% | 12.14% | 14.62% | 73.23% |  | 9.03% | 3.98% | 5.05% | 90.97% |
| Legume | 27.22% | 15.88% | 11.33% | 72.78% |  | 11.00% | 6.53% | 4.47% | 89.00% |
| Vegetable | 20.38% | 9.02% | 11.36% | 79.62% |  | 7.30% | 3.22% | 4.08% | 92.70% |
| Mushrooms | 29.17% | 16.53% | 12.64% | 70.83% |  | 16.09% | 9.16% | 6.93% | 83.91% |
| Fruit | 10.58% | 2.68% | 7.90% | 89.42% |  | 5.02% | 1.28% | 3.74% | 94.98% |
| Livestock | 29.78% | 16.65% | 13.12% | 70.22% |  | 11.92% | 6.68% | 5.23% | 88.08% |
| Poultry | 28.40% | 16.12% | 12.28% | 71.60% |  | 14.53% | 8.24% | 6.29% | 85.47% |
| Dairy | 16.56% | 8.72% | 7.84% | 83.44% |  | 9.40% | 5.02% | 4.38% | 90.60% |
| Egg | 19.52% | 10.17% | 9.34% | 80.48% |  | 7.60% | 3.98% | 3.61% | 92.40% |
| Fish | 23.38% | 13.94% | 9.44% | 76.62% |  | 10.94% | 6.54% | 4.40% | 89.06% |
| Snack | 38.86% | 34.76% | 4.10% | 61.14% |  | 22.89% | 20.22% | 2.67% | 77.11% |
| Fastfood | 14.67% | 5.74% | 8.93% | 85.33% |  | 9.05% | 3.73% | 5.32% | 90.95% |
| Beverage | 29.92% | 13.80% | 16.12% | 70.08% |  | 24.96% | 9.12% | 15.83% | 75.04% |
| Alcohol | 31.28% | 26.66% | 4.62% | 68.72% |  | 14.63% | 12.54% | 2.09% | 85.37% |
| Sugar | 26.85% | 14.81% | 12.04% | 73.15% |  | 15.43% | 8.51% | 6.91% | 84.57% |
| Average | 24.12% | 13.71% | 10.41% | 75.88% |  | 8.75% | 4.97% | 3.78% | 91.25% |

Summary: Results show that individuals eat more mushrooms, livestock, poultry and snacks, and drink more beverages and alcohol when they eat away from home. Vegetables, fruit, dairy, eggs and fast food are consumed less frequently away from home.
